# Demographics and Prevalence of Risk Factors in Patients With Peripheral Arterial Disease Presenting to a Tertiary Care Center in Northern India

**DOI:** 10.7759/cureus.32397

**Published:** 2022-12-11

**Authors:** Sawan Sharma, Venkata Vineeth Vaddavalli, Ajay Savlania, Ashwitha Ravi, Lileswar Kaman, Arunanshu Behera

**Affiliations:** 1 Department of General Surgery, Postgraduate Institute of Medical Education and Research, Chandigarh, IND

**Keywords:** prevalence, cardiac risk factors and prevention, chronic disease epidemiology, risk factors, peripheral arterial diseases

## Abstract

Introduction

Globally around 236.62 million people over 25 had the peripheral arterial occlusive disease (PAOD) in 2015, out of which 72.91 were in lower and middle-income countries. It will be a significant health problem in our country as the Indian population is aging. Our study aims to determine the demographics and prevalence risk factors in patients presenting with PAOD.

Methods

This prospective observational study was conducted from January 2020 to April 2021 at a tertiary care center in Northern India. A total of 275 patients with PAOD were enrolled in the study. The demographics and the prevalence of the risk factors were studied.

Results

Males (85.8%) were predominantly affected compared to females (14.2%). About 80.4% were smokers, of which 84.16% predominantly smoked Bidi. Around 39.3% had diabetes mellitus, 43.3% were hypertensive, and dyslipidemia was present in 17.1%. History of coronary artery disease and cerebrovascular accident was present in 22.3% and 12%, respectively. Farming was the occupation for 29.1% and 32.4% had not received formal schooling. The mean annual income was Rupees 1,56,025 (rupees 13,000 per month). Overall, the socioeconomic status was low.

Conclusion

The demographics and the prevalence of risk factors in patients with PAOD in Northern India differ from those in South India and the Western populations. Most of the patients were elderly males belonging to low socioeconomic status. There is a need for a central registry for vascular diseases in India to monitor the trends of the annual incidence of PAOD and to look for the prevalence of risk factors.

## Introduction

Peripheral arterial occlusive disease (PAOD) is an atherosclerotic disease predominantly involving the arteries of the lower limbs. Globally around 236.62 million people over the age of 25 years had PAOD in 2015, out of which 72.91 million were in lower and middle-income countries [1]. In India, as most of the population consists of older adults, it will be a significant health burden. The most common risk factors associated with atherosclerotic vascular disease are age, male gender, smoking, diabetes mellitus, hyperlipidemia, hypertension, and less commonly race (Western>Asian), hyperhomocystinemia, increased C-reactive protein, and chronic renal insufficiency [2]. The challenge for the vascular specialist is to recognize the presence of lower extremity ischemia, quantify the extent of local and systemic disease, determine the degree of func­tional impairment related to PAOD, and identify and control modi­fiable risk factors [3]. The data regarding the demographics and risk factors of patients with PAOD is limited in India [4]. In this study, we aim to assess the demographics and risk factors in PAOD patients presenting to a tertiary care center in Northern India.

## Materials and methods

This prospective observational study was conducted from January 2020 to April 2021 at the Post Graduate Institute of Medical Education and Research, Chandigarh, India. This study was approved by Institutional Ethical Committee (INT/IEC/2020/SPL-1082). Proper informed written consent was obtained from all the study participants. A total of 275 consecutive patients with PAOD were enrolled in this study. PAOD was defined as ankle-brachial pressure index (ABPI) <0.9. Patients diagnosed with Buerger's disease and Takayasu arteritis were excluded from the study. Data on demographics were collected, including age, sex, residence, occupation, education, and socioeconomic factors. Risk factors like smoking, diabetes mellitus, hypertension, dyslipidemia, dietary habits, and associated comorbid conditions of coronary artery disease and cerebrovascular disease were assessed. We defined diabetes mellitus as fasting blood sugar ≥126mg/dl, hypertension as systolic blood pressure ≥140 mm Hg and/or diastolic blood pressure ≥90 mm Hg, and/or on medication for hypertension. Dyslipidemia was defined as serum low-density lipoprotein (LDL) ≥130mg/dl, serum total cholesterol ≥200 mg/dl, and serum high-density lipoprotein (HDL) <40mg/dl in men or <50 mg/dl in women. Blood workup including Hemoglobin, hematocrit, erythrocyte sedimentation rate (ESR), C reactive protein, renal function test, lipid profile, fasting blood sugar, and glycosylated Hemoglobin (HbA1c) was done in all the patients. For those under the age of 50 years-serum homocysteine levels and thrombophilia workup (Fibrinogen level, Protein C, S, AT-III, prothrombin time (PT) and activated partial thromboplastin time (aPTT), Factor V Leiden, Lupus anticoagulant) was done. All the data regarding the patients were maintained in an electronic case record form. Data were coded and recorded in MS Excel spreadsheet program. SPSS version 23 (IBM Corp., Illinois, USA) was used for data analysis. Descriptive data were elaborated as means/standard deviations and medians for continuous variables and frequencies and percentages for categorical variables.

## Results

The median age (in years) of our study cohort was 58. 85.8% (236) of the participants were males, and 14.2% (39) were females. The data regarding the demographics are shown in Table 1.

**Table 1:** Demographics of our study cohort

Demographic	N(%)
Median age in years	58
Age	
≤50 Years	53 (19.3%)
51-60 Years	124 (45.1%)
61-70 Years	67 (24.4%)
71-80 Years	24 (8.7%)
81-90 Years	7 (2.5%)
Gender	
Male	236 (85.8%)
Female	39 (14.2%)
State	
Punjab	95 (34.7%)
Haryana	80 (29.2%)
Himachal Pradesh	62 (22.6%)
Uttar Pradesh	14 (5.1%)
Others	11 (4.0%)
Jammu & Kashmir	7 (2.6%)
Uttarakhand	5 (1.8%)
Education	
No Schooling	89 (32.4%)
Primary School	55 (20.0%)
Middle School	41 (14.9%)
High School	66 (24.0%)
Graduate	20 (7.3%)
Post-Graduation	1 (0.4%)
Professional	3 (1.1%)
Occupation	
Farmer	80 (29.1%)
Business/ Shopkeepers	44 (16.0%)
Housewives	35 (12.7%)
Retired Government Officials	29 (10.5%)
Government Officials	15 (5.5%)
Professional	5 (1.8%)
Unemployed	1 (0.4%)
Others	66 (24.0%)
Mean annual income	156025.45

About 80.4% of the study participants were smokers. 29.1% (80) of them were farmers by occupation. The laboratory workup is summarised in Table 2.

**Table 2:** Laboratory workup of the study population ESR: Erythrocyte sedimentation rate, CRP: C reactive protein, LDL: Low-density lipoprotein, HDL: High-density lipoprotein, FBS: Fasting blood sugar, PT: Prothrombin time, aPTT: Activated partial thromboplastin time

Investigations	Mean ± SD
Haemoglobin (g/dL)	10.88 ± 1.94
Hematocrit (%)	33.68 ± 10.37
Platelet Count (x10^3^/cu.mm)	275.76 ± 113.23
ESR (mm/Hr)	25.50 ± 3.70
CRP (mg/L)	16.80 ± 11.88
Blood Urea (mg/dL)	33.41 ± 15.97
Creatinine (mg/dL)	1.07 ± 2.34
Triglycerides (mg/dL)	144.07 ± 58.96
LDL (mg/dL)	68.44 ± 37.88
HDL (mg/dL)	37.22 ± 11.20
Total Cholesterol (mg/dL)	150.44 ± 50.41
FBS (mg/dL)	116.20 ± 33.73
HbA1c (%)	7.92 ± 0.58
PT (sec)	14.75 ± 0.79
aPTT (sec)	39.80 ± 11.69

The mean ABPI is 0.41 ± 0.20, with 55% of the participants having an ABPI of <0.4. In the risk factor assessment of the study group, 39.3% (108) had diabetes mellitus with a mean duration of 76.21 months.

Around 15.7% (17) of the participants were not taking any treatment for their diabetes, 75.0% (81) were on oral hypoglycemic agents, and 8.3%(9) were on Insulin therapy. A history of coronary artery disease was present in 22.3% (61) of the participants, and cerebrovascular disease in 12.0% (33) of the participants. The data regarding the risk factors and comorbid conditions are shown in Table 3.

**Table 3:** Risk factors and Comorbidities of the study population OHA: Oral hypoglycemic agents

Risk factor/Comorbidity	N(%)
Diabetes Mellitus	108 (39.3%)
Mean duration of Diabetes (months)	76.21
Duration of Diabetes	
<1 Year	21 (19.8%)
1-5 Years	38 (35.8%)
5-10 Years	29 (27.4%)
>10 Years	18 (17.0%)
Treatment for Diabetes	
None	17 (15.7%)
OHA	81 (75.0%)
Insulin	9 (8.3%)
OHA + Insulin	1 (0.9%)
Coronary artery disease	61 (22.3%)
Cerebrovascular accident	33 (12.0%)
Chewable Tobacco	4 (1.5%)
Smoking	221 (80.4%)
Form of Smoking	
Bidi	186 (84.1%)
Cigarette	23 (10.4%)
Cigarette + Bidi	12 (5.4%)
Mean pack years of smoking	40.49
Alcohol Use	178 (64.7%)
Dyslipidemia	47 (17.1%)
Obesity	32 (11.6%)
Hypertension	119 (43.3%)

## Discussion

Our study depicts the demographic profile and the prevalence of the risk factors of patients with PAOD in Northern India. Globally, aging combined with the growing prevalence of risk factors such as diabetes, smoking, hypertension, dyslipidemia, and obesity contributes to an increased prevalence of PAOD [5]. Increasing age is a significant risk factor associated with PAOD, and the median age of our study population is 58 years. In the western population, men and women appear approximately equally affected, and a study conducted in Beijing showed that women had a greater incidence of PAOD than men, which is similar to a study from Kerala, India [4,6,7]. However, most other studies have found PAOD prevalence more in men or equal in both sexes [8]. In the present study, males (85.8%) were more affected than females (14.2%). The majority (34.7%) of the patients were from the state of Punjab, and education-wise, most (32.4%) had not received any formal schooling, and farming was the occupation of the majority (29.1%). The mean annual income was Rupees 1,56,025 (rupees 13,000 per month), and the socioeconomic status was low overall.

About 80.4% of our study participants were smokers compared to 31% in a study from Southern India and 14-21% in the western population [4,9-11]. Interestingly, Bidi (a mini cigar filled with tobacco flakes and commonly wrapped in a tendu or Piliostigma racemosum leaf tied with a string or adhesive at one end) was the preferred form of smoking in 84.16% as compared to cigarettes. Although moderate alcohol consumption has been reported to be protective against PAOD, heavy alcohol consumption can be a potential risk factor for developing PAOD [12]. In our study, 64.7% of the participants consumed alcohol in varying quantities ranging from moderate to heavy consumption.

Hypertension is another significant risk factor for PAOD. In our study, 43.3% of the participants had hypertension compared to 62.9% from the data from South India and 29 to 47% in the western population [4,9-11]. Diabetes is another known risk factor for PAOD; 39.3% of the participants in our study had diabetes, and the figures from Kerala and the western studies were 25.5% and 10%, respectively [4,9-11]. Duration of diabetes is related to an increased incidence of PAOD, and elevated HbA1c is associated with the progression of PAOD; our study revealed a mean duration of diabetes in participants to be 76.21 months and mean HbA1c level of 7.92 as compared to South India, where it is 9.1years and an HbA1c of 9.1 [7]. Dyslipidemia was noted in 17.1% of patients in our study versus 61.6% prevalence in the study conducted in Kerala and 30 to 46% in the Rotterdam study and other western reports [4,9-11].

Subherwal and associates analyzed data from a large registry. They concluded that patients with PAOD had a higher risk of long-term cardiovascular events and suggested that PAOD should be considered a coronary artery disease (CAD) risk-equivalent [13]. The Kerala study reported 15% as having definite CAD [4]. The Edinburgh Artery Study observed CAD in 71% of individuals with symptomatic PAOD and 54% in asymptomatic subjects [14]. Other studies evaluating CAD in patients with proven PAOD report a prevalence of CAD between 50 and 70% [15,16]. Our study showed that 22.3% of the respondents had a history of CAD treated either medically or with PCI. Obesity is independently associated with the severity of PAOD [17]. Abdominal fat distribution, but not total body fat, is associated with PAOD [18]. In our study, 11.6% of the participants were obese (BMI > 30) vs. 29.06% in the Kerala study. 12% had a history of a cerebrovascular accident (CVA), and a study from Sri Lanka reported that 11.4% of patients with PAOD had a history of CVA in their population [8]. The data showing the prevalence of risk factors in our study as compared to other studies are summarized in Table 4.

**Table 4:** Comparison of prevalence of risk factors in our study with existing studies

	Risk factor	Our study	Kerala [4]	Sri Lanka [8]	Rotterdam study, Framingham offspring study, MESA [9,10,11]
1	Smoking	80.4 %	31%	44%	14-21%
2	Alcohol consumption	64.7%	-	48.9%	-
3	Hypertension	43.3%	62.9%	78.5%	29-47%
4	Diabetes mellitus	39.3%	25.5%	71.6%	10%
6	Coronary artery disease	22.3%	15%	15.9%	50-71%
7	Dyslipidemia	17.1%	61.6%	73.9%	30-46%
8	Cerebrovascular accident	12%	-	11.4%	-
9	Obesity	11.6%	29.06%	-	-

The majority of PAOD has been the subject of numerous investigations. The best method of assessing the prevalence of chronic lower extrem­ity arterial occlusive disease is to record the ABPI. A normal ABPI ranges between 0.9 to 1.2 [19,20]. The mean ABPI in our study was 0.4, indicating that most patients presented with chronic limb-threatening ischemia, reflecting a delayed presentation; possible reasons could be financial constraints, lack of medical facilities, and a lack of understanding about PAOD among the patients.

This study shows that the demographics and the prevalence of risk factors in the patients with PAOD in the northern part of India differ from those in Southern India and the Western population. As there is a growing incidence of PAOD in growing populations, with the majority constituting older adults like in India, there is a need for increased awareness of PAOD in the country. There is a need for a national registry that will help us better estimate the annual incidence of PAOD, demographics, and risk factors. Outreach camps to increase awareness of PAOD and to screen people for risk factors will help to reduce the incidence. The limitation of this study was the smaller sample size.

## Conclusions

The demographics and the prevalence of the risk factors for PAOD in our study differ from those in southern India and the western population. The majority were males in their late 50s, with smoking as a major risk factor. Most of the study population belongs to the lower socioeconomic status, which indicates the need for increased awareness in that population. Having a national registry for PAOD will help better estimate the demographics and prevalence of the risk factors and monitor the trend of incidence and prevalence over the years.
